# The difference of primary school teachers’ online teaching satisfaction in subject and educational level

**DOI:** 10.3389/fpsyg.2022.1027591

**Published:** 2023-01-04

**Authors:** Yonghai Zhu, Di Liu, Yingying Xu

**Affiliations:** ^1^Elementary Education Collage, Capital Normal University, Beijing, China; ^2^Sustech Education Group, Nanshan No.2 Experimental School, Shenzhen, China

**Keywords:** online teaching satisfaction, primary school teachers, subject, educational level, difference

## Abstract

The growth of online education requires high-quality online teaching. Teachers’ satisfaction with online teaching is of great significance for improving online teaching effectiveness. This study was to explore the primary school teachers’ online teaching satisfaction during the spread of COVID-19 from Shanghai, who have experienced online teaching, and explore whether there were differences of teachers’ online teaching satisfaction in subject and educational level. 939 teachers from Shanghai participated in the study. The non-parametric Mann–Whitney U test and Kruskal-Wallis test of variance were performed. Results showed that teachers’ online teaching satisfaction was at a high level. Moreover, there was a significant difference in teachers’ subject and educational level on online teaching satisfaction. In terms of subject differences of teachers, there were significant differences in resource suitability (RS) among teachers of different subjects. Therefore, it is recommended that each subject should develop the online teaching resources to support teachers’ online teaching. In terms of the differences in teachers’ educational levels, there were no significant differences between the satisfaction of college-level teachers on non-technical variables such as content selectivity (CS) and teachers with undergraduate and graduate degrees, and only on technical variables such as self-efficacy (SE), resource suitability (RS), ease of use (EU), and intention to use (IU). Given the national context in China, the difference in educational levels may be more reflected in the age of the teachers. For teachers with college educational level, due to their older age, rather than simply enhancing motivation and improving learning ability to increase online teaching satisfaction, emphasis should be placed on providing appropriate teaching service support to help improve online teaching effectiveness. The findings provide new empirical evidence, strategies and Chinese experience for promoting teachers’ online teaching satisfaction.

## 1. Introduction

The global face-to-face learning and teaching activities have been negatively impacted by the worldwide COVID-19 pandemic from the primary to college school levels (Catalano et al., 2021). In this context, online teaching has been the main method instead of traditional face-to-face learning (Khaldoun et al., 2021). A large number of students carried out all their learning activities online, prompting the teachers to design online courses to facilitate student learning (Evans, 2014). To promote better online learning effectiveness, higher-quality online teaching is also in demand (Kim and Freberg, 2018). Compared with the traditional teaching mode, online teaching has the advantages of breaking the limitations of teaching time and space, flexible teaching method organization, rich and convenient teaching resources (Zaikov et al., 2021). With a long period of online teaching practice during the COVID-19 lockdown, as the main participants and practitioners of online education, teachers’ satisfaction with online teaching is an important reflection of online teaching effectiveness (Liu and Zhang, 2021).

However, teachers will encounter many challenges in the process of switching from the traditional classroom teaching to online teaching (Russell, 2015). Teachers will have many problems in the transition to online teaching, such as being unsuited to new roles and dissatisfied with online teaching resources. In the process of transitioning to online teaching, teachers will become dissatisfied and disappointed with the responsibilities assumed by their new roles (Lemay et al., 2021). Some teachers still resist online teaching, believing that online teaching will reduce student participation and thus affect students’ academic performance (Damşa et al., 2021). Since the COVID-19 epidemic, the number of students participating in online learning has continued to increase, and students have a strong demand for online teaching, but teachers still have many problems facing online teaching (Cutri et al., 2020). Teachers’ satisfaction with online teaching is of great significance for improving the quality of online teaching.

So far, a large number of studies have explored the factors affecting online teaching, but there is a lack of evaluation and difference analysis of teachers’ satisfaction with online teaching. Therefore, the study clarifies the current status of teachers’ satisfaction with online teaching during the spread of COVID-19, which can help education managers design plans to improve teachers’ online teaching satisfaction. In China, the Shanghai Municipal Education Commission was the first metropolis to propose the full adoption of online education during the epidemic. More crucially, the Shanghai Municipal Education Commission proposed that the city’s key teachers be selected by the Commission to record unified online teaching videos for each grade level of basic education, each 20 min in length, and then provide them to the city’s teachers as basic through online teaching platforms, television and video distribution. Teaching resources. The district or school may decide to adopt all or part of it on its own. In this context, this current study analyzes the differences of teachers’ online teaching satisfaction in their different subject and educational levels, provides targeted development for teachers of different educational levels, and better promote the effect of students’ online learning.

## 2. Literature review

### 2.1. Teachers’ satisfaction with online teaching

As one of the four elements of the teaching system, the teacher plays a very important role and is the leader of the whole teaching process (Noben et al., 2021). Therefore, improve teachers’ satisfaction with online teaching is crucial. Teachers’ online satisfaction refers to teachers’ affective reactions to their teaching role or to their work (Sancar, 2009). Based on the American Customer Satisfaction Index model framework for customer satisfaction as a reference (Anderson et al., 1994), the factors influencing online teaching satisfaction for teachers were identified. There are also many factors have been proved to affect teachers’ satisfaction with online teaching. The teacher’s emotional state will affect the teacher’s work efficiency and job satisfaction (Sadeghi et al., 2021). Bolliger et al. (2014) pointed out that teachers’ satisfaction with online teaching is affected by factors, including reliable technology, workload, compensation, preparation, and evaluation. Online teaching requires teachers to have technical skills and access to online teaching resources (Chou and Chou, 2021). Orr et al. (2009) asserted that the teacher’s online teaching course preparation time is too long, which will affect the online teaching satisfaction. Faculty’s technological literacy has different influence on their faculty satisfaction with online teaching (Tabata and Johnsrud, 2008). Culp-Roche et al. (2021) stated that the higher online teachers self-efficacy, the more satisfied they are with online teaching. Self-efficacy is positively correlated with online teaching satisfaction (Horvitz et al., 2014). There was a study showed that teachers under the epidemic had lower self-efficacy when compared with the normative sample (Cataudella et al., 2021), which means teachers in epidemic situation had lower level of job satisfaction (Moè et al., 2010). The interaction between learners and teachers will affect the teaching effect of teachers, and then affect teachers’ satisfaction with online teaching (Tang, 2021). Factors related to teachers’ work can significantly affect teachers’ satisfaction with online teaching (Marasi et al., 2020). In addition to the factors mentioned above, there may be many other factors that affect teachers’ satisfaction with online teaching (Kara et al., 2021). Under the epidemic situation, Elshami et al. (2021) found that among teachers who participated in online teaching, they were generally satisfied with online teaching. This section briefly summarizes the relevant literature on factors affecting teachers’ online teaching satisfaction. These factors help this study explore the online teaching satisfaction of teachers during the COVID-19.

### 2.2. The role of the subject and educational level in teachers’ teaching

#### 2.2.1. Subject

The development of students is diversified, requiring not only the learning of scientific and cultural knowledge, but also the development of personal expertise and interests (Roth and Roychoudhury, 1993). In the information age, students’ learning is personalized. In addition to completing the core subjects prescribed by the state, they can also choose other special subjects according to their abilities and interests (Hsu et al., 2017). In the Chinese primary schools, all subjects are divided into two types of subjects: core and special. Core subjects include Chinese, Math and English, and special subjects include Music, Art, and Physical education (PE; Xie and He, 2017). The core subjects help students learn scientific and cultural knowledge and exercise their minds, while the special subjects help students develop their personal expertise and expand their interests. The core subjects help students learn scientific and cultural knowledge, while the special subjects help students develop their personal interests and expertise (Korkeamäki and Dreher, 2011). Sadeghi et al. (2021) explores the difference in job satisfaction between English teachers and non-English teachers. They showed that no significant difference, but compared with English teachers, non-English teachers are more experienced. The characteristics of teachers will be affected by subject knowledge, certification and experience (Mills et al., 2020). Teachers’ teaching self-efficacy is directly affected by personal interests and indirectly affected by subject knowledge (Ekstam et al., 2017). Nortvig et al. (2020) showed that different situations in which teachers of the Art, Craft, and Design use blended learning. Arbaugh (2013) conducted a two-year experiment to study the relationship between subject differences and online courses, and the results showed that subject differences would affect teachers’ teaching effectiveness and satisfaction. Albert et al. (2021) explored whether there are subject differences among teachers of different subject types in terms of course models, instructional tools, and teaching methods in an online teaching environment. Different subjects have different knowledge attributes, and teachers of each subject will consider subject factors to design online teaching courses (Badia and Gomez, 2014). Kember and Kwan (2000) showed that subject and curriculum have different degrees of influence on online teaching approaches. However, through online teaching research, Gonzalez (2009) found that teachers of different subjects have little influence on the methods and effects of online teaching. For online teaching, the relationship between subject and online teaching satisfaction is still uncertain. Therefore, this study explored the differences in satisfaction with online teaching among teachers of different subjects.

#### 2.2.2. Educational level

Educational level refers to the highest level of education that teachers receive. Teachers’ educational level will affect teachers’ job satisfaction, and there are significant differences in teachers’ job satisfaction with different education levels (Abu Taleb, 2013). Teachers with higher education levels have higher computational and digital literacy (Tafazoli et al., 2017), and they are more likely to master the operational techniques and pedagogy of online teaching. Teachers’ education level has an impact on their perception of online teaching platform functions and platform operability, which can affect teachers’ satisfaction and willingness to continue using online teaching (Liu et al., 2021). Teachers’ school level and education level can influence online teachers’ self-efficacy and e-learning readiness. Sherrod (2014) research showed that people with a higher education level are positively correlated with providing high-quality services, which could also suggest that teachers with higher levels of education provide higher quality online education. Lim (2009) proposed that significant difference was found in teachers’ job satisfaction based upon the level of teachers’ degree of education. Less-educated teachers tended to have more positive attitudes and thus used more technology in class in comparison to more educated teachers (Arar and Abramovitz, 2017). During the COVID-19 epidemic, teachers also faced the technical pressure of online teaching, and teachers of different educational levels had different levels of teaching technology (Chou and Chou, 2021). Demirtas (2010) measured the job satisfaction of primary school teachers, and the results showed that no significant difference existed in professional seniority. Teachers with lower educational levels are more active in online teaching, which may be because they lack specific teaching techniques and solid knowledge compared with teachers with higher educational levels (Diep et al., 2016). However, when it comes to whether there is a relationship between teachers’ online teaching satisfication and educational level during the epidemic, there are very few studies and certain conclusions. Therefore, this study explored the relationship between teachers’ educational level and online teaching satisfaction during the COVID-19 epidemic.

### 2.3. Research questions

In order to narrow the research gap, this current study aims to explore teachers’ satisfaction with online teaching during the COVID-19 epidemic from the perspective of teachers’ subject and educational level.

In this study, three research questions were posed:

1. How satisfied are primary school teachers with online teaching in each dimension of an online teaching satisfaction framework?
2. Are there significant differences in teachers’ satisfaction with online teaching in terms of teachers’ teaching subject?
3. Are there significant differences in teachers’ satisfaction with online teaching in terms of teachers’ educational level?

## 3. Methodology

### 3.1. Participants

The participants of the study were teachers from primary schools who carried out online education during the COVID-19 epidemic in Shanghai, China. The questionnaire was sent to a platform for online survey powered by Questionnaire Star^1^ which is an open and free website, and distributed to the participants in Shanghai, China. In total, 939 samples were collected in the questionnaire, 30 incomplete questionnaires were deleted from the data and 909 completed questionnaires were finally analyzed to report the results (see Table 1). Among 909 teachers, there were 566 core subject teachers (62.3%) and 343 special subject teachers (37.7%). There were 103 teachers with college education level (11.3%), 734 teachers with undergraduate degree (80.7%) and 72 teachers with post-graduate degree (7.9%). Among them, there are 100 male teachers (11.1%) and 809 female teachers (88.9%).

**Table 1:** Demographic characteristics of the participants.

	Variable	Frequency	Percent (%)
Subject	Core	566	62.3
	Special	343	37.7
Education level	College	103	11.3
	Undergraduate	734	80.7
	Post-graduate	72	7.9
Gender	Male	100	11.1
	Female	809	88.9

Junior college graduate (i.e., College) is a level of education in China. College students have 3 years of schooling, 1 year less than the undergraduate degree. In 2022, the statistics of the Ministry of Education of China show that the undergraduate enrollment rate of senior high schools in China is about 42%, while the enrollment rate above junior college is over 92%.

### 3.2. Instruments

Based on the American Customer Satisfaction Index (ASCI) model (Anderson et al., 1994), combined with the factors affecting teachers’ online teaching satisfaction obtained by literature analysis, a measurement tool is constructed. In addition, reliability and validity tests were also carried out to ensure the accuracy and validity of the questionnaire. The questionnaire consisted of five sub-scales, content selectivity (CS), resource suitability (RS), self-efficacy (SE), ease of use (EU) and intention to use (IU), which are reflected in two categories: non-technical variables (CS) and technical variables (RS, SE, EU, and IU).

The researchers in this paper believe t that CS refers to teachers’ choice of learning content (Zhu et al., 2022). In terms of CS, Lee and Park (2008) version of teacher selection teaching content was used. This questionnaire was adapted from the questionnaires of other scholars (Davis, 1989; Davis et al., 1989; Pavlou, 2003) to investigate the choice of teaching content. Since their research is aimed at consumers rather than teachers, we further revised the questionnaire. Specifically, “Teachers can choose learning content,” “Teachers can adjust the details of the learning content,” and “Teachers can adjust the difficulty of the learning content.”

RS refers to the teachers’ specific choice and control of the learning resource, especially electronic media resources or online digital resources, which has great influence on the course quality and fruitfulness (Zhu et al., 2022). In terms of RS, using the revised version of teachers’ intention to use online education resources by Chang et al. (2010), the resource suitability for teachers in Shanghai was investigated. For instance, “After simple editing, the teaching resources provided online can be well applied to my teaching,” and “The materials of internet teaching resources are the same as the teaching materials I use.”

The difference between this study and previous studies is the addition of the CS variable and the distinction of CS from RS in previous studies on satisfaction. The teaching system consists of four elements: teachers, students, content, and media (resources), and RS and CS represent the “content” and “media (resources)” of the four elements, respectively. They are non-technical variables and technical variables respectively, so the difference between them is that RS focuses on “the form of resources,” “the fit of resources and materials,” and “the usability of resources “CS and RS have a different focus because the Shanghai Municipal Education Commission provides unified, basic learning content and video resources, and different district education departments, schools, or grassroots teaching and research groups may have different requirements for teachers; or use different media such as online teaching or live TV, which may impose constraints on teachers and ultimately result in teachers being able or not being able to. choose their own teaching content based on the content and resources provided by the Shanghai Education Commission.

Self-efficacy refers to the teacher’s teaching self-efficacy (Linnenbrink and Pintrich, 2002). Teaching self-efficacy or the sense of teaching efficacy means the confidence of teachers to promote the growth and development of students and the development of values (Tschannen-Moran et al., 1998). In this study, the questionnaire was modified in combination with the scale of Huang et al. (2019), and the definition of self-efficacy (Li, 2019), and five questions were formed. One of the questions was deleted because of its low validity. For example, “I can master a variety of communication tools.” and “I can carry out and exit various applications smoothly.”

Ease of use refers to teachers are supported by various technical equipment and environment in teaching (Compeau and Higgins, 1995). Teaching technology equipment and environment include hardware devices such as computers, projectors, and tablets, and software devices such as online teaching platforms and software applications, and so forth (Luca et al., 2019). In this study, the questionnaire was modified in combination with the scale of Huang et al. (2019), and the definition of ease of use for teachers (Compeau and Higgins, 1995). Finally, the following items were retained: “The school equipment internet is sufficient and can meet my use requirements at any time,” “The school gives me sufficient permission to use the online teaching resources needed for teaching,” and “When I want to use the online teaching resources, I will not delay my use due to equipment, the internet or other reasons.”

Intention to use refers to the way teachers are willing to use online teaching. The questionnaire developed by Wu and Short (1996) was modified in this study to assess teachers’ intention to use. Teachers’ high intention to use online teaching indicates that teacher’ satisfaction with online teaching is relatively high. Items were retained: “I am willing to use online teaching resources frequently,” “I am willing to try more online teaching methods in teaching,” and “After the COVID-19 epidemic, I will continue to use online teaching if I can.”

To verify the applicability of the scale, a pre-survey was conducted in this study. The items were analyzed using high and low grouped independent sample t-tests, and items with poor discriminatory power were eliminated based on decision values (Zhu et al., 2022). The final questionnaire consists of 5 sub-scales, with a total of 16 items, including 3 items of CS, 4 items of SE, 3 items of RS, 3 items of EU and 3 items of IU. These five sub-scales roughly reflect all dimensions of online education satisfaction. The answers of the questionnaire were rated on a 5-point Likert scale (1 = very dissatisfied, 2 = relatively dissatisfied, 3 = average, 4 = relatively satisfied, 5 = very satisfied). Filter out missing and invalid data and re-moved them, SPSS26.0 and Amos26.0 software were used for data analysis. Internal consistency test and confirmatory factor analysis were performed to ensure the reliability and validity of the instrument. The results show that the questionnaire has high internal consistency. The overall Cronbach’s alpha of 0.930 and 0.903 (CS), 0.844 (SE), 0.845 (RS), 0.743 (EU), and 0.846 (IU) for each sub-scale. The structure validity of the scale was judged. The KMO value was 0.930, and the Barlett sphere test reached a significant level (*p < 0*.*001*). Confirmatory Factor Analysis showed that the questionnaire had acceptable psychometrical properties (*x^2^/DF = 2*.*754; p < 0*.*001; RMSEA = 0*.*053; GFI = 0*.*950; NFI = 0*.*936; CFI = 0*.*958; IFI = 0*.*958; TLI = 0*.*946*). According to the model fit indices criteria (Byrne, 2006), the model fit statistics show that the online teaching satisfaction model is a perfect fit.

### 3.3. Procedure

The study was conducted with the consent of the school principal and teachers. The questionnaire was sent to a platform for online survey powered by Questionnaire Star (see text footnote 1) which is an open and free website, and distributed to the participants in Shanghai, China. Before completing the questionnaire, teachers were provided with brief instructions on how to complete the questionnaire and the required time. All the teachers were informed that their answers were anonymous and the results of the questionnaire could be applied in a publication.

### 3.4. Data analysis

SPSS 26.0 was utilized in the study. The descriptive and inferential statistics were calculated. Firstly, teachers’ satisfaction with online teaching is analyzed by calculating the means and standard deviation. Then, graphical techniques and the Shapiro–Wilk test (*p > 0*.*05*) are used to check whether the original data conforms to the normal distribution (Shapiro and Wilk, 1965). The results showed that the original score data of the dependent variables such as CS, SE, RS, EU, and IU did not accord with the normality hypothesis. Therefore, non-parametric the Mann–Whitney U test and Kruskal-Wallis test were used for the difference analysis (Corder and Foreman, 2009; Roever and Phakiti, 2018).

The Kruskal-Wallis test is a non-parametric test equivalent to analysis of variance (ANOVA), and is used to test the overall hypothesis that there is no significant differences among two or more groups based on the mean ranks (Pallant, 2002; Hazra and Gogtay, 2016). The Kruskal-Wallis test aims to determine whether there were significant differences in online teaching satisfaction among the three different educational levels (i.e., college, undergraduate, post-graduate). Independent variables were subject and educational level, dependent variables were the five sub-scales of online teaching satisfaction. Finally, a Kruskal-Wallis post-hoc pairwise comparison of samples showed that there were significant differences between samples (banneheke et al., 2017).

## 4. Results

### 4.1. Online teaching satisfaction

The means and standard deviations of teachers’ online teaching satisfaction were shown in Table 2. The average degree of satisfaction is greater than 3.5, indicating that teachers’ online teaching satisfaction was at the medium level. Among the five sub-scales, the teachers scored the highest in CS with a mean of 4.43 (*SD = 0*.*62*), followed by SE (*M = 4*.*11*, *SD = 0*.*67*), RS (*M = 4*.*15*, *SD = 0*.*66*), EU (*M = 4*.*11*, *SD = 0*.*74*), and IU (*M = 4*.*01*, *SD = 0*.*76*).

**Table 2:** Overall satisfaction of teachers of different subject and educational level.

Satisfaction *M* (SD)	Subject *M* (SD)		Educational level *M* (SD)		
	Core (*n* = 566)	Special (*n* = 343)	College (*n* = 103)	Undergraduate (*n* = 734)	Post-graduate (*n* = 72)
CS 4.43 (0.62)	4.43 (0.62)	4.41 (0.61)	4.41 (0.67)	4.44 (0.60)	4.35 (0.75)
SE 4.11 (0.67)	4.14 (0.66)	4.07 (0.69)	3.84 (0.71)	4.14 (0.66)	4.21 (0.68)
RS 4.15 (0.66)	4.22 (0.64)	4.05 (0.70)	4.01 (0.65)	4.18 (0.67)	4.12 (0.60)
EU 4.11 (0.74)	4.14 (0.74)	4.07 (0.75)	3.94 (0.77)	4.14 (0.74)	4.1 1(0.70)
IU 4.01 (0.76)	4.02 (0.75)	3.99 (0.76)	3.73 (0.81)	4.05 (0.74)	3.98 (0.73)

### 4.2. Mathematics academic achievement

#### 4.2.1. Subject

The follow-up non-parametric Mann–Whitney U test with subject IV showed that teachers were CS (*U = 95094*.*500*, *z = −0*.*546*, *p = 0*.*585*, *Cohen’s d = 0*.*034*), SE (*U = 91595*.*500*, *z = −1*.*444*, *p = 0*.*149*, *Cohen’s d = 0*.*095*), RS (*U = 84342*.*000*, *z = −3*.*391*, *p = 0*.*001*, *Cohen’s d = 0*.*221*), EU (*U = 92209*.*000*, *z = −1*.*287*, *p = 0*.*198*, *Cohen’s d = 0*.*084*) and IU (*U = 93816*.*000*, *z = −0*.*860*, *p = 0*.*390*, *Cohen’s d = 0*.*056*; see Table 3). In statistics, *p < 0*.*05* is generally considered to represent a statistically significant difference, and *p = 0*.*001 < 0*.*01* in the RS dimension, indicating that there is a significant difference between teachers’ subject and online teaching satisfaction in the dimension of RS. Moreover, the effect size Cohen’s d is generally considered in statistics as a small effect when it is 0.2 to 0.5, a medium effect when it is 0.5 to 0.8, and a large effect when it is greater than 0.8, indicating that the RS dimensions where the differences are located are all small effects. When mean rank points are investigated, it appeared the core subject teachers are generally higher than the special subject teachers. Table 3 provides a summary of mean ranks for participants, U values, Z values, and significance levels.

**Table 3:** Summary of Mann–Whitney U test statistic.

Independent variable	Dependent variable	Mean ranks		*U*	*Z*	*P*
		Core	Special			
Subject	CS	458.49	459.46	95094.500	−0.546	0.585
	SE	464.67	439.04	91595.500	−1.444	0.149
	RS	477.49	417.90	84342.000	−3.391	0.001
	EU	463.59	440.83	92209.000	−1.287	0.198
	IU	460.75	445.52	93816.000	−0.860	0.390

#### 4.2.2. Educational level

The results of the Kruskal-Wallis test analysis are shown in Table 4, indicating that there were significant statistical differences among the three groups of teachers. Among the five sub-scales of online teacher satisfaction, teachers’ SE (*x^2^ = 19*.*206*, *p = 0*.*000*, *η^2^ = 0*.*019*), RS (*x^2^ = 7*.*395*, *p = 0*.*025*, *η^2^ = 0*.*006*), EU (*x^2^ = 6*.*493*, *p = 0*.*039*, *η^2^ = 0*.*005*), IU (*x^2^ = 14*.*922*, *p = 0*.*001*, *η^2^ = 0*.*014*), CS (*x^2^ = 0*.*244*, *p = 0*.*885*, *η^2^ = 0*.*002*). Statistical *p*-values were obtained based on the significance test, generally *p* < 0.05 for statistically differences, *p* < 0.01 for statistically significant differences, and *p* < 0.001 for extremely significant statistical differences, with the smaller the p-value the more significant the difference (Lancaster and McQueeney, 2011). This shows that the three groups of teachers have extremely significant differences in the SE dimension, significant differences in the IU dimension, and just differences in both RS and EU. Moreover, the effect size η^2^ (Eta-squared) is generally considered in statistics as a small effect at 0.01, a medium effect at 0.06, and a large effect at 0.14, indicating that all four dimensions in which the differences are located are small effects. In the CS dimension, the three groups of teachers did not have any differences. When the rank mean points related to educational level and teachers’ online teaching satisfaction are investigated, it showed that teachers with the undergraduate educational level (456.59) are higher than those with the college educational level (452.95) and post-graduate educational level (441.74) in terms of CS. However, it appeared that teachers with the college level are generally lower than teachers of undergraduate and post-graduate’ level in terms of SE, RS, EU, and IU.

**Table 4:** Summary of Kruskal–Wallis test statistic.

Independent variable	Dependent variable	Mean ranks			χ^2^	*df*	*P*
		College	Undergraduate	Post-graduate			
Educational level	CS	452.95	456.59	441.74	0.244	2	0.885
	SE	353.20	464.74	501.36	19.206	2	0.000
	RS	394.40	465.47	434.92	7.395	0.2	0.025
	EU	394.66	463.85	451.13	6.493	2	0.039
	IU	363.55	468.51	448.06	14.922	2	0.001

Based on the results of the Kruskal-Wallis test, paired *post hoc* two-way comparisons between the three groups of teachers also needed to be continued. The results of the *post hoc* two-by-two comparison are shown in Table 5, where there was an extremely significant difference between the college group and the undergraduate group in terms of SE and IU (*p = 0*.*000 < 0*.*001*), and the analysis also showed statistically significant differences between college and undergraduate in terms of RS (*p = 0*.*026 < 0*.*05*) and EU (*p = 0*.*033 < 0*.*05*). The smaller the value of p, the more significant the difference. Thus, the largest differences between the college and undergraduate groups were reflected in the SE and IU dimensions, followed by the RS dimension, and the smallest differences were in the EU dimension. In addition, according to the analyzed data, a significant difference was also found between the college group and the post-graduate group in the SE dimension (*p = 0*.*001 < 0*.*01*).

**Table 5:** Pairwise multiple comparisons for educational level.

Educational level			*P*
SE	College	Undergraduate	0.000
	College	Post-graduate	0.001
	Undergraduate	Post-graduate	0.759
RS	College	Undergraduate	0.026
	College	Post-graduate	0.913
	Undergraduate	Post-graduate	1.000
EU	College	Undergraduate	0.033
	College	Post-graduate	0.465
	Undergraduate	Post-graduate	1.000
IU	College	Undergraduate	0.000
	College	Post-graduate	0.100
	Undergraduate	Post-graduate	1.000

## 5. Discussion

The purpose of this study is to understand the satisfaction of primary school teachers with online teaching. It is hoped that it will help to further understand the satisfaction of teachers with online teaching and provide enlightenment for online teaching in the future.

### 5.1. Overall level of online teaching satisfaction among primary school teachers

The conclusion shows that teachers’ satisfaction with online teaching is generally high. Among them, teachers have the highest mean value in CS and RS, and lower frequency in SE and EU. According to the study of Marasi et al. (2020), faculty were satisfied with online teaching, which was consistent with the findings of this study. The study of Elshami et al. (2021) showed that most teachers were satisfied with online teaching. The consistency of findings contributes to a deeper understanding of overall satisfaction with online teaching in the context of the covid-19 pandemic. Teachers’ high satisfaction with online teaching is reflected in CS and the RS. Orr et al. (2009) asserted that establishing comprehensive online course resources can shorten the time for teachers to prepare for online teaching courses and improve their teaching efficiency. Teachers have high-quality online course resources, which will improve their emotional state. Sadeghi et al. (2021) showed that the teacher’s emotional state will affect the teacher’s work efficiency and job satisfaction. During the epidemic period, schools all over the country organized teachers to conduct online teaching and provided complete teaching resources so that teachers could conduct online teaching smoothly (Mishra et al., 2020). At the same time, having a wealth of online teaching content can allow teachers to conduct effective online teaching during the epidemic, and more recognize online teaching (Kara et al., 2021). Besides, teachers’ SE is an important factor affecting online teaching satisfaction. Participants in the study of Hampton et al. (2020) showed relatively high levels of online teaching satisfaction and online teaching self-efficacy. Teachers’ self-efficacy can be promoted to online teaching satisfaction. Bandura (1977) found that self-efficacy is critical. People with high self-efficacy are more willing to try to change their work environment and stick to work while facing with negative outcome expectations. Teachers with a high sense of self-efficacy can promote online teaching satisfaction to a high level (Lee, 2010). A number of studies have shown that, regardless of the individuals’ skill levels, self-efficacy is independently related to the achievement of goals (Bandura, 1977; Tschannen-Moran et al., 1998). Horvitz et al. (2014) found that maintaining a high sense of self-efficacy is very important for teachers, because in the face of negative outcome expectations and experiences, teachers with higher self-efficacy are more likely to persist. In addition, EU is also an essential part of online teaching satisfaction. Possess good educational technology and the skills to acquire teaching resources, which can enhance teachers’ online teaching satisfaction (Bolliger et al., 2014). The underlying factors for teachers’ dissatisfaction with online teaching are probably due to fear of network technology and disappointment in organizational support services (Sword, 2012). Therefore, paying attention to providing planned and targeted training for teachers of different educational levels can help reduce fear of online teaching, increase confidence in online teaching, and improve teaching quality and online teaching satisfaction.

### 5.2. Subject difference in teachers’ satisfaction with online teaching

Teachers of different subjects are generally satisfied with online teaching satisfaction, and there is no significant difference. Gonzalez (2009) found that there was little correlation between subject and online teacher teaching satisfaction. This coincides with the findings of this study. Specific to the five dimensions of online teaching satisfaction, teachers of different subjects have significant differences in RS, but there are no significant differences in other aspects. This is mainly due to the different knowledge attributes of different subjects and the difficulty for teachers of each subject to find online teaching resources (Badia and Gomez, 2014). Particularly before China’s “double reduction” policy, it was difficult to teach special subjects online in elementary school, which required more direct instruction from teachers to develop students’ individual strengths and interests, and therefore online teaching resources were scarce. However, teachers of special subjects had relatively few teaching tasks, especially during the epidemic, and online teaching was more compressed, so teachers had more time to prepare lessons and find resources online, which did not reduce teaching satisfaction. At the aspect of teaching resources, the core subject teachers have more teaching tasks than the special subject teachers, so the core subject teachers are looking for more online teaching resources (Sadeghi et al., 2021). The core subject teachers have more experience, so they are more proficient in acquiring teaching resources. In schools, online teaching resources for core subjects are relatively complete. Teachers can use these teaching resources conveniently to improve teaching effects. Teachers have a strong sense of self-efficacy, so they believe that they can find high-quality resources and carry out online teaching design to achieve good online teaching effect (Horvitz et al., 2014). Ekstam et al. (2017) found that there are more teaching trainings for the core subject teachers, and teachers have a stronger sense of self-efficacy, so they have better access to online teaching resources. Bolliger et al. (2014) pointed out that having reliable teaching technology, strong self-efficacy and perfect curriculum resources can reduce teachers’ workload and affect teachers’ satisfaction with online teaching. High-quality online teaching resources are the necessary conditions for teachers to conduct online teaching. Therefore, schools should establish comprehensive teaching resources, and allow teachers the freedom to choose and adapt learning content, for the core and special subjects to help teachers prepare for online teaching curriculum resources. Carry out teacher training in a planned and targeted manner, improve teachers’ educational technology ability, and enhance the ability to search online teaching resources, improve their sense of self-efficacy. It helps to improve the teaching quality of teachers and enhance the recognition of online teaching.

### 5.3. Educational level difference in teachers’ satisfaction with online teaching

Teachers’ online teaching satisfaction is also affected by teachers’ educational level. The satisfaction of teachers with the college educational level is significantly lower than teachers of undergraduate and post-graduate’ level, especially in terms of SE, RS, EU, and IU, while there is no significant difference between teachers with undergraduate level and teachers with post-graduate level. Lim (2009) found that there is statistically significant difference in teachers’ job satisfaction based upon the level of teachers’ educational level. A significant difference of teacher’s online teaching satisfaction was found in their education level. Teachers with a high level of education are more suitable for online teaching (Abu Taleb, 2013). There is a positive correlation between educational level and the provision of quality services (Sherrod, 2014). Therefore, teachers with higher education levels are more able to provide high-quality online teaching. A consistent conclusion is helpful for understanding the findings of this study. Specifically, in terms of SE, there are significant differences between teachers with post-graduate and undergraduate’s educational levels and teachers with college educational level. Teachers with a post-graduate’s level of education have a strong sense of self-efficacy, followed by teachers with a undergraduate’s level of education, and college educational level teachers have the lowest sense of self-efficacy. Teachers with college educational level have low self-efficacy and intention to use for online teaching because of their educational level (Sherrod, 2014). Teachers with undergraduate and post-graduate have a higher level of education, are easier to accept new teaching methods, and have a higher sense of self-efficacy in online teaching. The higher the self-efficacy of teachers, the better the organization and work planning (Allinder, 1994). Teachers’ sense of self-efficacy helps to improve work efficiency and job satisfaction. There are significant differences in aspects of RS, EU and IU, mainly manifested in the different proficiency of the information technology mastered by teachers. Teachers with a undergraduate’s degree are at the highest level, followed by teachers with a post-graduate’s degree, and teachers with a college’s degree are the lowest. Teachers of different educational levels face different technical pressures, their proficiency in mastering the technology is different, and the teaching resources they acquire are also different (Chou and Chou, 2021). Teachers of different educational levels have different technical pressures, which will affect teachers’ online teaching satisfaction. Tafazoli et al. (2017) believe that teachers with a high level of education have higher technical capabilities and information literacy. Teachers with undergraduate and post-graduate’s educational levels have higher digital literacy than teachers with college educational level. Teachers of different education levels have different effects on teaching learning (Yu, 2021). Teachers with a post-graduate’s degree have better teaching effects than college. In view of the educational technology pressure and insufficient self-efficacy faced by teachers of different educational levels, targeted training should be carried out to enhance their online teaching capabilities.

## 6. Conclusion

This study explored the online teaching satisfaction of primary school teachers during the COVID-19 epidemic in Shanghai, and analyzed the differences in teacher’s subject and educational level in online teaching satisfaction. The results show that primary school teachers in Shanghai are generally satisfied with online teaching. In terms of CS, SE, RS, and EU, it is at a high level of satisfaction. There are significant differences between teachers’ subject and online teaching satisfaction at the level of RS, but there are no significant differences in the other four sub-scales. Teachers of core subjects are generally more satisfied with online teaching than teachers of special subjects. Teachers’ educational level and online teaching satisfaction have significant differences in SE, RS, EU, and IU. The results showed that the online teaching satisfaction of the college teachers is generally lower than that of teachers at the undergraduate and post-graduate level.

### 6.1. Implications

For online teaching, the results of this study are expected to provide some teaching enlightenment for teachers and managers. Firstly, from the perspective of the relationship between the subject and online teaching satisfaction, more attention should be paid to the online teaching resources for each subject. For each subject, it is necessary to develop more online teaching resources, so that teachers of different subjects can selectively use the online teaching resources of the subject, so as to improve the effect of online teaching and improve the online teaching satisfaction of teachers of various subjects. Secondly, in view of the differences of teachers’ satisfaction with online teaching in different educational level, more guidance strategies tailored to the characteristics of teachers at different levels should be provided. Professional guidance is positively correlated with teacher job satisfaction (Chung and Kowalski, 2012). Considering age, teachers with college educational level are dissatisfied with online teaching and should pay special attention to enhance their technical support services. The CS satisfaction of teachers is the highest among the five variables, and there is no significant difference in variable level with the difference in education level. Obviously, it cannot be concluded that the low level of education leads to low satisfaction. In the context of China’s national conditions, in the past 10–20 years, in the recruitment of teachers in Shanghai’s metropolises, all of them have bachelor’s degree or above, and there will be a number of teachers with college degrees who are over 45 years old. Therefore, among Chinese teachers, the age level is more deeply reflected behind the educational level. For teachers at higher age levels, strategies cannot be simply put forward by “strengthening skill learning” and “strengthening learning motivation.” Thirdly, this study summarizes the main dimensions of measuring teachers’ online teaching satisfaction, and compiles a reliable questionnaire for measuring online teaching satisfaction, which provides a reference and contribution to the measurement of teachers’ online teaching satisfaction.

### 6.2. Limitations and future works

However, this study has some limitations. First, the teachers participating in the survey come from specific regions, and the results are limited to target regions, individuals and groups. Therefore, a large sample size should be considered in the future. Secondly, this study only uses the quantitative method of questionnaire, while qualitative methods (such as interview) may better understand the changes of teachers’ satisfaction with online teaching in different stages. Thirdly, the distribution of teachers in this study is uneven in subject and educational level. In view of the above limitations, qualitative research methods can be used to enrich the data and findings in future research. This study only conducted a cross-sectional study, and longitudinal or intervention studies can also be conducted to further explore teachers’ satisfaction with online teaching. In addition, in order to better summarize the research results, teachers in more regions should be involved to obtain a larger sample size.

## Data availability statement

The raw data supporting the conclusions of this article will be made available by the authors, without undue reservation.

## Ethics statement

Ethical review and approval was not required for the study on human participants in accordance with the local legislation and institutional requirements. Written informed consent for participation was not required for this study in accordance with the national legislation and the institutional requirements.

## Author contributions

All authors contributed to the conception of the idea, implementing and analyzing the experimental results, writing the manuscript, and read and approved the final manuscript. All authors contributed to the article and approved the submitted version.

## Funding

This study was funded by the National Education Science “Fourteenth Five Year Plan” 2022 Key Topic of the Ministry of Education “Research on the effective behavior system of dual-teacher classroom teaching in the context of high-quality and balanced education” (DCA220455).

## Conflict of interest

The authors declare that the research was conducted in the absence of any commercial or financial relationships that could be construed as a potential conflict of interest.

## Publisher’s note

All claims expressed in this article are solely those of the authors and do not necessarily represent those of their affiliated organizations, or those of the publisher, the editors and the reviewers. Any product that may be evaluated in this article, or claim that may be made by its manufacturer, is not guaranteed or endorsed by the publisher.

## Footnotes

^1^ www.wjx.cn
